# The Power of Strategic Social Media Influencer Communication to Improve Black Women’s Knowledge and Awareness of Environmental Endocrine-Disrupting Chemicals: Surveys of Instagram Users

**DOI:** 10.2196/66128

**Published:** 2025-07-25

**Authors:** Elissia T Franklin, Katherine E Boronow, Jennifer Liss Ohayon, Aleyana Momplaisir, Jenell Steele, Camille Kathleen Smith, LaShannon Taylor, Breanna D Brock, Korie A Grayson, Kalere Edgecombe, Brittany S Harris, Kristen N Pender, Ijeoma B Kola, Adana AM Llanos, Dede K Teteh-Brooks, Lilly Marcelin, Julia Green Brody, Robin E Dodson

**Affiliations:** 1 Silent Spring Institute, 320 Nevada Street, unit 302, Newton, MA, 02460, United States, 1 6173324288, 1 6173324284; 2 Resilient Sisterhood Project, Cambridge, MA, United States; 3 Triple G, LLC, Baltimore, MD, United States; 4 STEM So(ul)cial, Philadelphia, PA, United States; 5 Department of Biology, Alabama A&M University, Huntsville, AL, United States; 6 Department of Sociology, Stony Brook University, Stony Brook, NY, United States; 7 (Re)Defining the Image of STEM, LLC, Ann Arbor, MI, United States; 8 Princess Margaret Hospital, Public Hospital Authority, Nassau, Bahamas; 9 The New Era Creative, Atlanta, GA, United States; 10 Polecology, LLC, Petersburg, FL, United States; 11 University of Notre Dame, Notre Dame, IN, United States; 12 Cohort Sistas, Newark, DE, United States; 13 Department of Epidemiology, Mailman School of Public Health, Columbia University Irving Medical Center, New York, NY, United States; 14 Herbert Irving Comprehensive Cancer Center, Columbia University Irving Medical Center, New York, NY, United States; 15 Department of Health Disparities Research, University of Texas MD Anderson Cancer Center, Houston, TX, United States

**Keywords:** Black women, consumer products, endocrine-disrupting chemicals, environmental justice, environmental health literacy, intended behaviors, product options in women-engaged research, social media influencers, personal care products, chemical exposure, health literacy, social media, women, Instagram, awareness, product-use behaviors

## Abstract

**Background:**

Black women are disproportionately affected by hormone-related health conditions, which may result from higher exposures to endocrine-disrupting chemicals (EDCs) in consumer products. EDCs are chemicals that interfere with the body’s natural hormones.

**Objective:**

The Product Options in Women-Engaged Research project was developed to educate Black women about EDCs. We assessed the impact of strategic social media influencer (SMI) communication on knowledge and awareness of EDCs and intentions to change product-use behaviors.

**Methods:**

We recruited 7 SMIs to engage with their audiences about EDC-related information on Instagram. The SMIs attended a workshop to learn about EDCs in consumer products and then created Instagram content to share with their audiences. We surveyed SMIs at baseline and 1 month after they shared EDC-related content. SMI audiences were surveyed cross-sectionally before and after the SMIs posted EDC-related social media content. We evaluated social media engagement and analyzed the impact of these communications on SMIs and their audience.

**Results:**

The social media posts reached over 16,000 accounts and elicited over 28,000 engagements (eg, views, likes, and shares). SMIs’ EDC knowledge and awareness increased after attending the workshop and sharing newly created content, and the SMIs had greater intentions to avoid EDCs at follow-up than at baseline. Engagement with the social media content about EDCs also led to positive outcomes among SMI audiences and particularly impacted intentions to engage in exposure reduction behaviors. In total, 80% of follow-up survey respondents reported that, in the future, they will always consider a company’s chemical policy (n=68) and product ingredients when shopping (n=73) compared to 26.8% (n=63) and 46.9% (n=115), respectively, of baseline survey respondents who already reported doing so (*P*<.001). More follow-up respondents than baseline respondents self-reported an intention to avoid parabens (n=33, 32.7% vs n=39, 15.3%; *P*<.001), bisphenol A (n=25, 24.8% vs n=38, 14.9%; *P*=.03), per- and polyfluoroalkyl substances (n=17, 16.8% vs n=9, 3.5%; *P*<.001), and fragrance (n=6, 5.9% vs n=5, 2.0%; *P*=.08).

**Conclusions:**

Our findings demonstrate that strategic SMI partnerships incorporating a culturally tailored training program can be used to reach large audiences of Black women, improve knowledge about EDCs, and promote intentions to change behaviors to reduce exposures to EDCs.

## Introduction

Women from historically minoritized racial and ethnic groups face disproportionate exposures to environmental chemicals from consumer products compared to White women [1-7]. This is significant because exposures to consumer product chemicals, including endocrine-disrupting chemicals (EDCs), have been linked to adverse health outcomes that disproportionately affect Black individuals [8-13]. For example, Black women in the United States use a greater number of hair care products and intimate care products compared to other groups of women [1-3,14-16], and these products often contain numerous EDCs [17]. Black women also have higher rates of many hormone-mediated conditions, including uterine fibroids [18], infertility [19], and some aggressive forms of breast, ovarian, and endometrial cancers, especially those linked to poorer long-term outcomes [20-28], and these conditions may be linked to consumer product exposures [9,24-27,29]. In addition, Black women have higher rates of postoperative complications upon treatment for some of these conditions [30-33], as well as higher cancer-related death rates [21-23,28,34], underscoring the importance of exposure mitigation efforts.

Individual behaviors such as avoiding products with specific ingredients on product labels are an effective way to reduce exposure to EDCs [35-39]. Previous studies indicate that enhancing environmental health literacy among individuals—especially concerning the chemicals in personal care products, their regulation, and possible health risks—may lead to the avoidance of specific personal care products or ingredients [40]. For example, in a biomonitoring study, respondents took actions to reduce EDC exposure (eg, removing nonstick cookware) after receiving information about EDCs in a study report [41]. Behavioral changes were similar among respondents who received personal biomonitoring reports and those who only received study-wide results, indicating that broadly sharing information about EDCs can be effective at promoting behavior change. Providing individuals with information about how EDCs can affect health and how to avoid them may motivate and support informed decision-making about product choices.

Social media is a particularly promising communication tool for health communications and advancing health equity through evidence-based knowledge sharing. Social media is widely accessed, including by those in hard-to-reach communities, and has emerged as a powerful tool to raise awareness about health issues and promote healthy behaviors [42,43]. Social media influencers (SMIs) play a crucial role on social media platforms, shaping their audience’s attitudes and behaviors through engaging content, endorsements, and perceived credibility [44-46]. SMIs are especially known for sharing content curated for a specific audience, often with a consistent theme, so are poised to deliver culturally relevant health messages [47]. The authenticity of SMI content is a key driver of health behavior change among audiences and has been leveraged across industries, from marketing to nonprofit organizations [44,46,48,49]. Powell and Pring [44] synthesized extant literature to show that SMI posts play a critical role in the health perceptions and behaviors of their audience. SMIs and health promotion messages, however, have not traditionally focused on reducing exposures to EDCs, underscoring the importance of evaluating the effectiveness of SMIs in promoting environmental health.

To effectively reach Black women with health information, it is crucial that the messaging is both culturally relevant and community-tailored [47,50]. Thus, SMI content designed specifically for Black women may be a particularly effective strategy for disseminating information on EDCs. Notably, more than 80% of Black individuals report using at least 1 social media platform [51]. While there has been increasing recognition of the disparities in EDC exposure among Black women in the United States, there are relatively few studies that explore actionable solutions and intervention strategies.

Theoretical frameworks guiding SMI engagement in health communication remain limited [44]. To address this gap, we applied Borchers and Enke’s [52] general conceptual model for strategic communication through SMIs, which consists of 6 key phases: planning, influencer selection and outreach, preparation and coordination, content production, content distribution, and evaluation [49,53]. A central feature of this method is outsourcing communication management to the SMIs, who serve as brokers for stakeholder relationships and foster dialogue, trust, and authenticity. Strategic SMI communication is used by nonprofit organizations to generate awareness for their topics, expand audience reach, and sustain existing engagement [[49]]. Additionally, our approach draws on the community health worker (CHW) model, which trains community members to promote health and serve as knowledge mediators. CHWs are especially effective in delivering messages in a culturally competent and accessible way [47], and this model has been used for decades to address environmental health disparities. Our work represents a novel application that combines strategic SMI communications (as outlined by Borchers and Enke [52]) with the CHW approach, engaging SMIs as digital knowledge mediators of EDC-related knowledge.

We launched the Product Options in Women-Engaged Research (POWER) project in 2021 to increase knowledge and promote positive behavioral change around EDCs among Black women. Our initial work focused on engaging Black women in Massachusetts, and we learned that our community members often rely on digital resources, particularly SMIs, to learn about consumer products before making purchasing decisions. With this insight, we began partnering with SMIs to share accessible, culturally relevant, and high-quality information about EDCs in consumer products. Given that consumer product use is a modifiable source of EDC exposures, innovative research translation to Black consumers about EDCs in their products is needed to inform decisions and reduce exposure disparities. The purpose of this study was to evaluate the effectiveness of partnering with SMIs to improve EDC-related knowledge, awareness, and behaviors.

## Methods

### Overview

The POWER project is a community-research partnership between the Resilient Sisterhood Project, a nonprofit organization that supports women of African descent on reproductive health issues; Resilient Sisterhood Project community members; Silent Spring Institute, a nonprofit organization that conducts environmental health research; and SMIs. We used strategic SMI communication to engage a large audience of Black women with EDC-related information. The objective was to improve EDC knowledge, awareness, and behavior by working with SMIs as knowledge mediators.

### Survey Development

To measure whether our strategic SMI communication improved knowledge and intentions for behavior change, we used surveys to evaluate EDC knowledge, awareness, and behaviors before and after engagement with EDC-related information on social media. We adapted the survey used by Boronow et al [41] to assess the responses of SMIs and their followers. The baseline survey consisted of 5 sections: knowledge of EDC exposures and regulations, awareness of common EDCs, individual behaviors (eg, shopping behaviors and chemical avoidance), demographics, and contact information. The knowledge section asked 6 questions that assessed understanding of EDC exposures and regulations (Table S1 in Multimedia Appendix 1). Respondents could answer true, probably true, probably false, and false. The EDC awareness section consisted of 3 yes-no questions asking respondents if they had heard of per- and polyfluoroalkyl substances (PFAS), bisphenol A (BPA), and parabens separately. Respondents who answered “yes” to having heard of a specific EDC were prompted to select a description of their use in consumer products from multiple options. We asked 4 individual questions in the behavior section to determine respondents’ actions at baseline (ie, before exposure to our program, Table S2 in Multimedia Appendix 1). Three questions asked about the frequency of behaviors (always, often, sometimes, rarely, and never) in the past year. The fourth question was open-ended to evaluate the chemicals that respondents were avoiding. The follow-up survey was the same as the baseline survey, excluding the demographic questions and 1 question in the individual behavior section. We also modified the behavior section to ask about respondents’ intended behaviors in the future. All survey questions were optional and could be skipped by respondents.

### SMI Recruitment, Training, and Baseline Survey

We targeted the recruitment of nano- and microinfluencers (<100,000 followers) because they tend to have relatively high engagement to follower count on Instagram (ie, on average, their like count for each post was at least 3% of their follower count) compared to influencers with larger reach [54]. We predicted that this would increase the likelihood of followers taking the surveys. To find SMIs who identified as Black women and served an audience predominantly of Black women, we asked for recommendations among our study partners and used Instagram’s recommendations. We contacted SMIs by email describing the project, why the SMI would be a good fit, and compensation.

We held digital preparation and coordination workshops with the contracted SMIs to teach them about the health effects of EDCs, disproportionate exposures among Black women, and evidence-based methods to reduce exposures (see Table S3 in Multimedia Appendix 1 for an overview of workshop topics). We also provided instructions for preparing their draft social media posts and sending them for review. Each SMI attended one of two 60-minute workshops with the study team and other SMIs. The SMIs completed the baseline survey at the start of the workshop. The workshop presentation was tailored to support the integration of survey content into the SMIs’ posts. For example, the survey included a question that asked whether everyday products—like shampoo, sofas, and plastic bottles—sometimes contain chemicals that can upset the balance of hormones in a person’s body. We thus suggested as a talking point that SMIs mention “chemicals that disrupt a person’s hormones are in everyday products including cosmetics, furniture, cookware, and plastic food and drink containers” (see Table S1 in Multimedia Appendix 1 for survey questions and suggested talking points). Within 1 week of attending the training workshop, SMIs shared a link on their Instagram account inviting their audience to take the baseline survey. All study surveys were available through Qualtrics using SMI-specific links, and respondents confirmed that they were at least 18 years of age. Baseline survey responses were collected in September and October 2023. Follow-up survey responses were collected in October and November 2023.

### Content Creation, Follow-Up Survey, and Post Analytics

After completing the training workshop, SMIs drafted social media content based on what they learned. We reviewed the developed content for scientific accuracy only; the editorial style of posts reflected each SMI’s personal interests, voice, and social media presence. SMIs distributed the approved content to their Instagram with a link to the follow-up survey. SMIs also completed the follow-up survey during this time.

To evaluate engagement, SMIs shared analytics from their Instagram accounts with the study team at least 30 days after the content was posted, including the number of views, accounts reached, likes, comments, and shares. We manually recorded all public comments from each post to a spreadsheet. SMIs were compensated US $1500.

### Analysis

Using an a priori list of talking points presented at the SMI training workshops (Table S1 in Multimedia Appendix 1), we reviewed the content posted by the SMIs and documented specific topics for subsequent analysis.

The SMI survey responses were analyzed separately from the responses of their audiences. We summarized the demographics of the SMIs shared in the baseline survey. Respondents could select all race and gender categories that applied. If a respondent selected Black as one of multiple categories, the respondent was categorized as Black. We compared SMIs’ individual responses at baseline and follow-up for all sections of the survey.

For the survey responses of SMIs’ audiences, we analyzed baseline and follow-up surveys as independent, cross-sectional samples because we were unable to track respondents to match their responses to the 2 surveys. We summarized audience demographics from the baseline survey, including mean age and SD. For each question in the knowledge section, true or false responses were coded as correct or incorrect, and probably true and probably false responses were coded as correct but unsure or incorrect but unsure. We compared baseline responses to the knowledge questions by demographic group using the Fisher exact test.

We then organized the questions from the knowledge section based on how many SMIs included the relevant content information in their posts. For questions that were covered by all the SMIs or none of the SMIs, we compared the proportion of respondents who answered the question correctly at baseline and follow-up. To more precisely assess question content covered by some but not all SMIs, we distinguished follow-up respondents as “viewers” and “nonviewers” and analyzed their responses separately. We analyzed each question by comparing follow-up survey responses among respondents who saw a post that addressed a question (viewers) to respondents who saw a post that did not cover the question (nonviewers). We identified viewers as respondents who responded to the Qualtrics survey link specific to an SMI who shared content that covered a specific question. For instance, if content addressing a question was mentioned by 5 SMIs, we compared the responses of the audiences for those 5 SMIs to the responses from audiences from the other 2 SMIs. We also evaluated awareness of BPA, PFAS, and parabens (eg, “Have you heard of parabens before?”) among baseline and follow-up survey respondents and whether respondents could correctly select the description of the EDC (eg, “Which of these statements is true for parabens?”).

We compared the differences between the proportion of baseline respondents and follow-up respondents who answered “always” or “often” to the questions about the frequency of behaviors. In the baseline survey, we also summarized responses to how often they bought a product with worrisome chemicals because they could not afford a safer one. We compared the proportion of respondents on the baseline and follow-up surveys who freely listed PFAS, parabens, fragrances, and BPA as chemicals to avoid. We use the term “chemical” to refer to PFAS, parabens, fragrances, and BPA based on the wording of the question. To compare responses of baseline and follow-up surveys by SMI audiences, we applied the Fisher exact test to determine statistical significance (α=.05). Data were processed and analyzed using R (version 4.3.1; R Foundation for Statistical Computing).

### Ethical Considerations

This study was reviewed and granted an exemption from further review by Northeastern University’s institutional review board (protocol IRB#23-05-10 Franklin). All procedures adhered to the ethical standards of the institutional review board and were consistent with the principles outlined in the World Medical Association’s Declaration of Helsinki. All participants self-reported that they were 18 years of age or older and provided informed consent electronically before participating in the web-based surveys. Data collected from surveys were deidentified prior to analysis, and no personally identifying information was reported. Responses were stored on secure, password-protected servers accessible only to authorized members of the Silent Spring Institute research team. Survey participants did not receive compensation.

## Results

### SMI Characteristics and Thematic Analysis of Their Social Media Posts

We onboarded 7 SMIs. In total, 4 SMIs attended the first workshop and 3 attended the second workshop. All the SMIs were identified as Black women and had at least a bachelor's degree. All but 1 lived in the United States, and the mean age of SMIs at baseline was 30.6 (SD 5.7) years. Their social media niches included art, wellness, hair, food, fashion, lifestyle, and sustainability, and SMIs had follower counts ranging from 5930 to 54,800 accounts on Instagram at the time of the campaign (Table S4 in Multimedia Appendix 1). SMIs developed content based on topics that interested them from the training workshop, including ingredients to avoid, health disparities, and the presence of EDCs in everyday products. The SMIs posted their content on Instagram using videos or carousels (posts with multiple images or videos that are viewed by swiping left or right).

**Figure 1.**
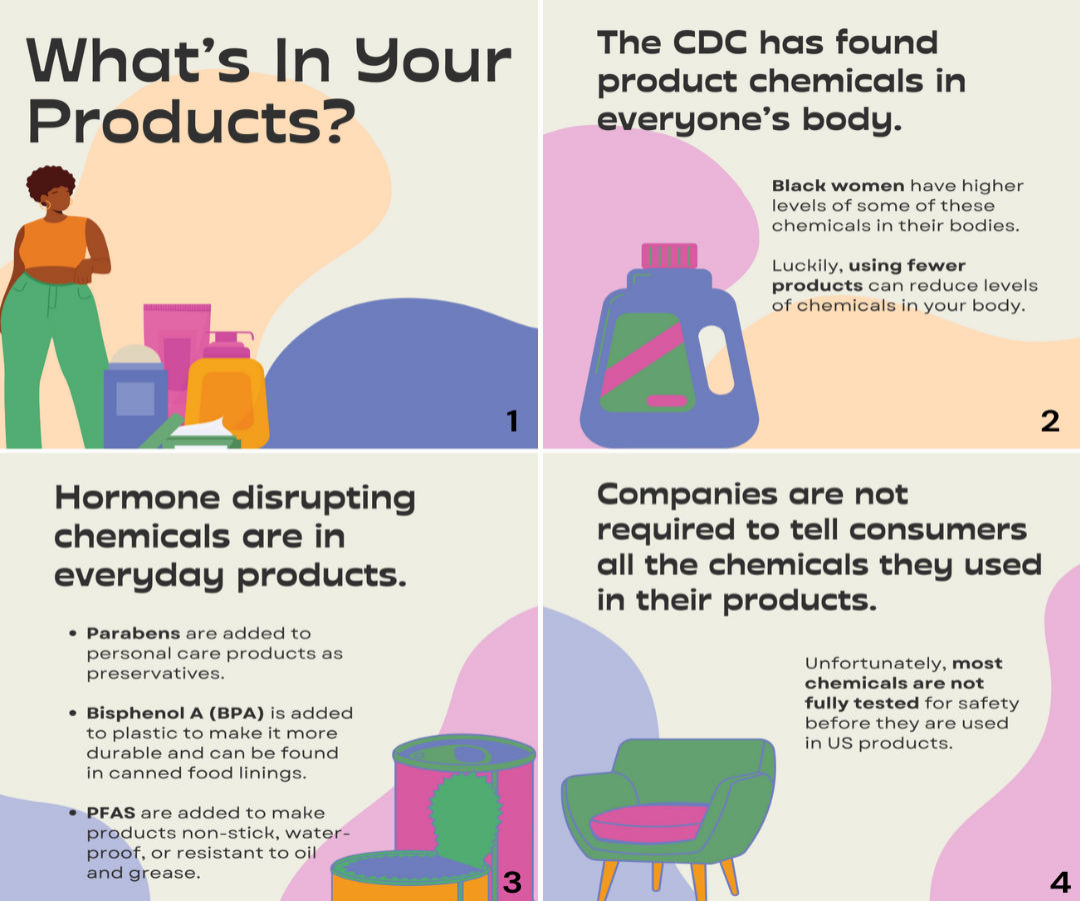
Instagram post created by Brittany Harris (@beeharris), a Product Options in Women-Engaged Research project social media influencer. CDC: Centers for Disease Control and Prevention; PFAS: per- and polyfluoroalkyl substance; US: United States.

SMIs shared evidence-based information about toxic chemicals in consumer products while incorporating audience appeal based on the SMI’s knowledge about their audience. For example, @beeharris shared a carousel where she incorporated the talking points about environmental justice issues from the training workshop (Figure 1). She shared tips on how to reduce chemical exposures and encouraged her audience to engage with the content in her caption, which read:

Hormone disrupting chemicals may be in the products that you use every day. Here are some ways to avoid them.

- Look for paraben-free products.
- Eat more fresh food instead of packaged foods.
- Opt for stainless and glass instead of plastic water bottles.
- Stay clear of products advertised as stain-resistant.
- What are ways you avoid chemicals in your products?

In total, 4 of the 7 SMIs mentioned environmental justice issues, specifically that Black women had higher levels of personal care product chemicals in their bodies compared to other groups of women. A total of 5 of the SMIs mentioned health conditions associated with EDCs. All SMIs mentioned at least 3 of the talking points shared at the workshop (Table S1 in Multimedia Appendix 1). The only knowledge talking point mentioned by every SMI was a statement that everyday products sometimes contain chemicals that can upset the balance of hormones. All the SMIs mentioned BPA, 6 mentioned parabens, and 5 mentioned PFAS in their content. Collectively, the SMI content reached over 16,000 accounts and garnered over 27,000 views. The posts also gained over 1100 likes and over 100 comments, shares, and saves (Table 1). The overall engagement by reach (ie, sum of likes, comments, shares, and saves divided by the number of accounts reached) was 10%.

**Table 1.** Total engagement of the social media influencer campaign.

Metrics	Values, n
Views (includes multiple views per account)	27,418
Unique accounts reached	16,186
Likes	1167
Comments	172
Shares	127
Saves	144

### SMI EDC Knowledge

**Figure 2.**
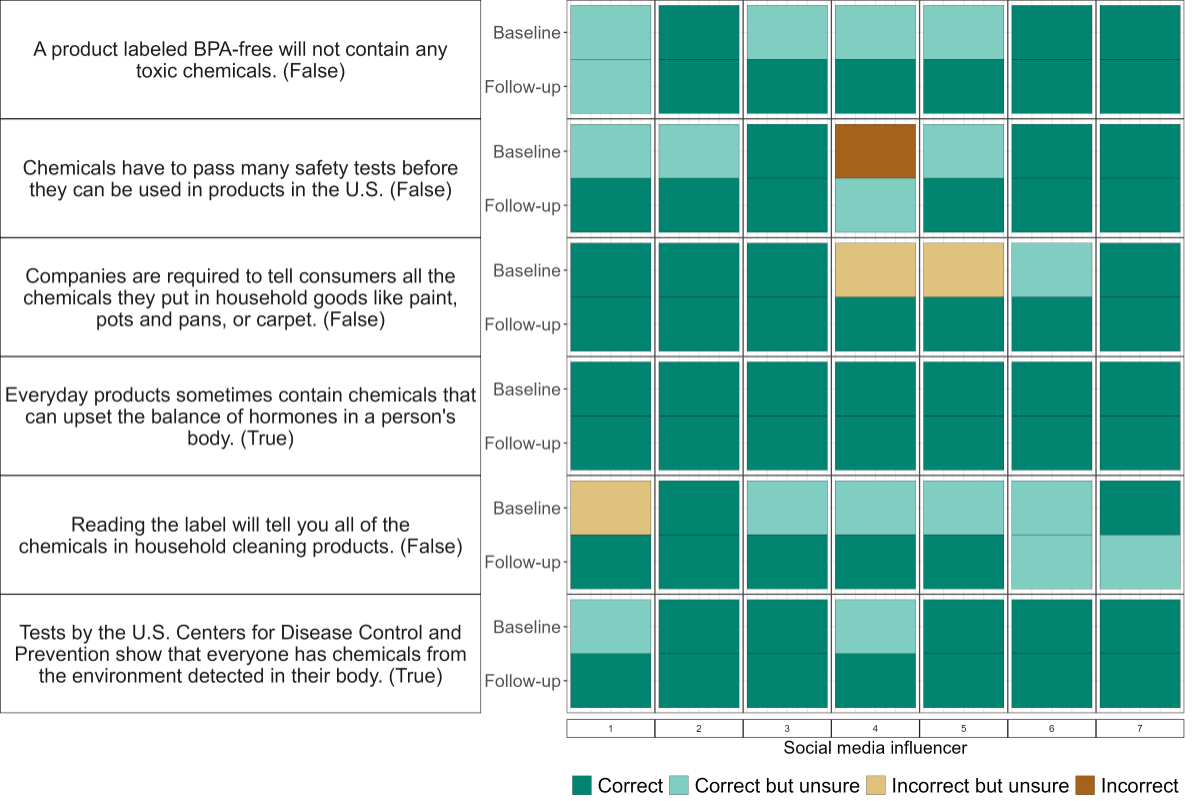
Baseline and follow-up survey responses of the 7 social media influencers (labeled at bottom) for questions in the endocrine-disrupting chemical knowledge section. Correct answer shown in parentheses. BPA: bisphenol A; US: United States.

SMI knowledge was high at baseline, with 6 of the 7 SMIs providing 0 or 1 incorrect response (Figure 2).

All SMI responses were correct on the follow-up survey, and only 4 of those responses were unsure, indicating greater confidence in knowledge after participation. Responses to the follow-up showed improvement in 16 of the 18 cases when the baseline response was not correct.

### SMI EDC Awareness

**Figure 3.**
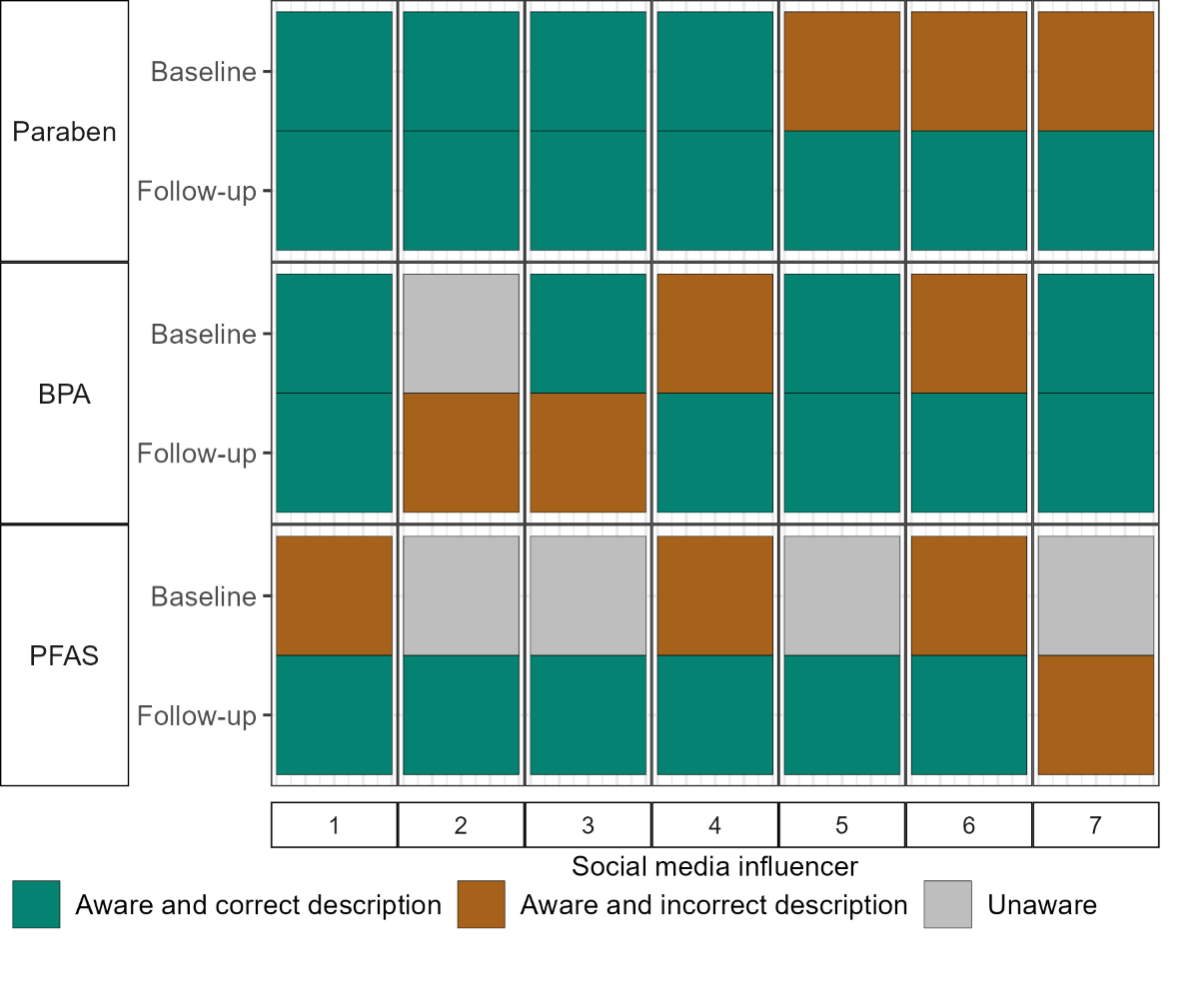
Baseline and follow-up survey responses of social media influencers for EDC awareness questions. For social media influencers who indicated that they had heard of an EDC before, they were then asked to select its description. BPA: bisphenol A; EDC: endocrine-disrupting chemical; PFAS: per- and polyfluoroalkyl substance.

At baseline, SMIs had greater awareness of parabens and BPA compared to PFAS (Figure 3), but, for all 3 chemical types, several SMIs did not know their uses. Awareness of all 3 chemical groups improved at follow-up, with 100% of SMIs correctly identifying how parabens are used.

### SMI Behaviors

A greater number of SMIs reported avoiding specific ingredients at follow-up compared to baseline. A total of 3 SMIs reported avoiding parabens, BPA, PFAS, or fragrances at baseline, while 6 SMIs avoided 2 or more of these chemicals at follow-up (Figure S1 in Multimedia Appendix 1). Only 1 influencer did not list a chemical to avoid in the follow-up.

In total, 4 SMIs reported at baseline that they often or always check a product label for chemical ingredients before they buy it, and this increased to 6 at follow-up (Figure S2 in Multimedia Appendix 1). At baseline, all SMIs reported that they sometimes or often considered a company’s policy when purchasing, and this increased to often or always for all SMIs at follow-up.

### Audience Demographics

A total of 255 respondents recruited by SMIs took the baseline survey: 82.4% (n=210) of the respondents identified as women, and 68.2% (n=174) identified as Black (Table 2). In total, 64.3% (n=164) of the respondents identified as Black women, confirming our ability to reach our target audience. The respondents had a high level of formal education, with 69.0% (n=176) having a bachelor’s degree or higher. A total of 101 respondents took the follow-up survey.

**Table 2.** Self-reported characteristics of social media influencers’ audiences who responded to the baseline survey^a^.

Characteristics	Respondents (N=255)
Age (years)	
Mean (SD)	32.3 (8.6)
Missing, n (%)	36 (14.1)
Race, n (%)	
Black^b^	174 (68.2)
Non-Black	44 (17.3)
Missing	37 (14.5)
United States resident, n (%)	
Yes	204 (80)
No	28 (11)
Missing	23 (9)
Gender, n (%)	
Woman	210 (82.4)
Woman, nonbinary	1 (0.4)
Nonbinary	3 (1.2)
Man	11 (4.3)
Missing	30 (11.8)
Education, n (%)	
Up to high school graduate	8 (3.1)
Some college	31 (12.2)
Technical school, trade school, or associate degree	13 (5.1)
Bachelor degree	83 (32.5)
Graduate degree	93 (36.5)
Missing	27 (10.6)

a Proportions may not sum to 100% due to rounding.

b Respondents could select all race and gender categories that apply. If Black was selected as one of multiple categories, the respondent was categorized as Black.

### Audience EDC Knowledge

**Figure 4.**
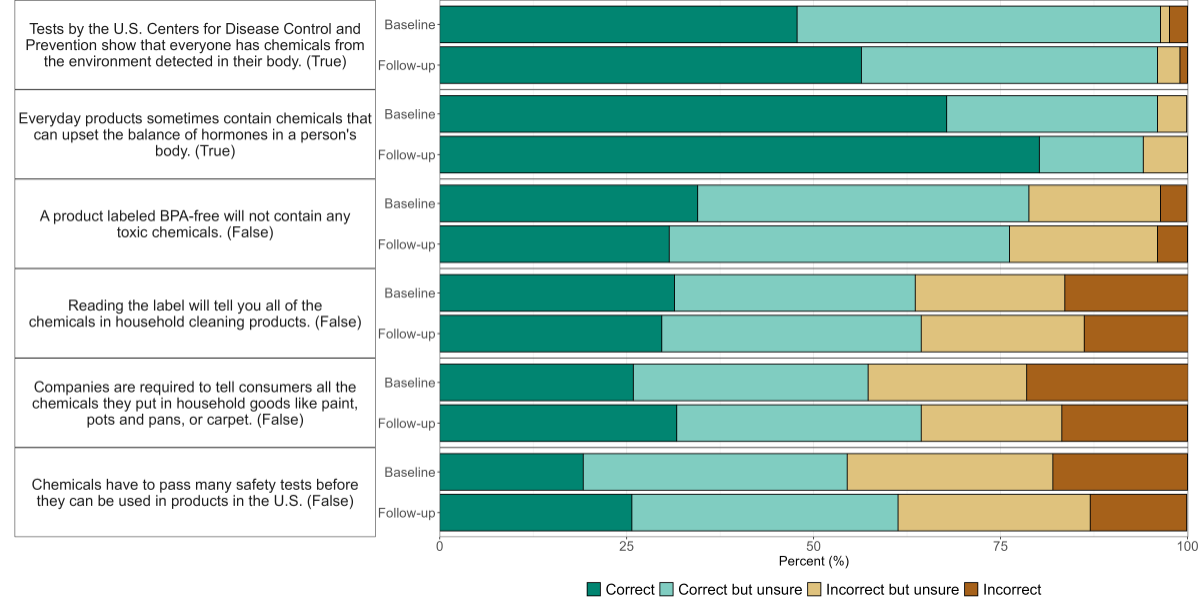
Baseline (N=255) and follow-up (N=101) survey responses from audiences for endocrine-disrupting chemical knowledge section. Questions are ordered by descending percentage of correct and correct but unsure at baseline. Correct answer shown in parentheses. BPA: bisphenol A.

For all questions at both baseline and follow-up, more than half of the survey respondents answered correctly or correctly but unsure (Figure 4). At baseline, the highest proportion of correct responses (n=173, 67.8%) was for the question that everyday products sometimes contain chemicals that can upset the balance of the hormones in a person’s body (true), and an additional 28.2% (n=72) of responses were correct but unsure. The lowest proportion of correct responses (n=49, 19.2%) was for the question that chemicals have to pass many safety tests before they can be used in products in the United States (false), and an additional 35.3% (n=90) answered correctly but unsure. Respondents also had difficulty with the question that companies are required to tell consumers all the chemicals they put in household goods like paint, pots and pans, or carpet (false, n=66, 25.9% correct).

There were some differences in the proportion of correct responses by demographic group at baseline (Table S5 in Multimedia Appendix 1). A significantly higher percentage of Black women compared to non-Black women correctly answered the question that everyday products contain chemicals that can upset the balance of hormones in a person’s body (n=118, 72.0% vs n=29, 54.7%; *P*=.02, Fisher exact test). A greater percentage of respondents with a bachelor's degree or higher compared to those with an associate degree or lower correctly answered the questions that companies are required to tell consumers all the chemicals they put in household goods (n=53, 30.1% vs n=9, 17.3%; *P*=.08) and the question that reading the label will tell you all of the chemicals in cleaning products (n=61, 34.7% vs n=11, 21.2%; *P*=.09).

Significantly more follow-up survey respondents (N=101) answered that everyday products sometimes contain chemicals that can upset the balance of hormones in a person’s body correctly compared to baseline respondents (n=81, 80.2% vs n=173, 67.8%, respectively; *P*=.02, Table S6 in Multimedia Appendix 1 and Figure 4). As expected, there was no difference in baseline and follow-up survey responses for questions based on content not mentioned by any SMI in their posts. For each knowledge answer mentioned by a subset of SMIs, a greater proportion answered correctly among those who viewed a relevant post compared to those who did not (Table S7 in Multimedia Appendix 1).

### Audience EDC Awareness

**Figure 5.**
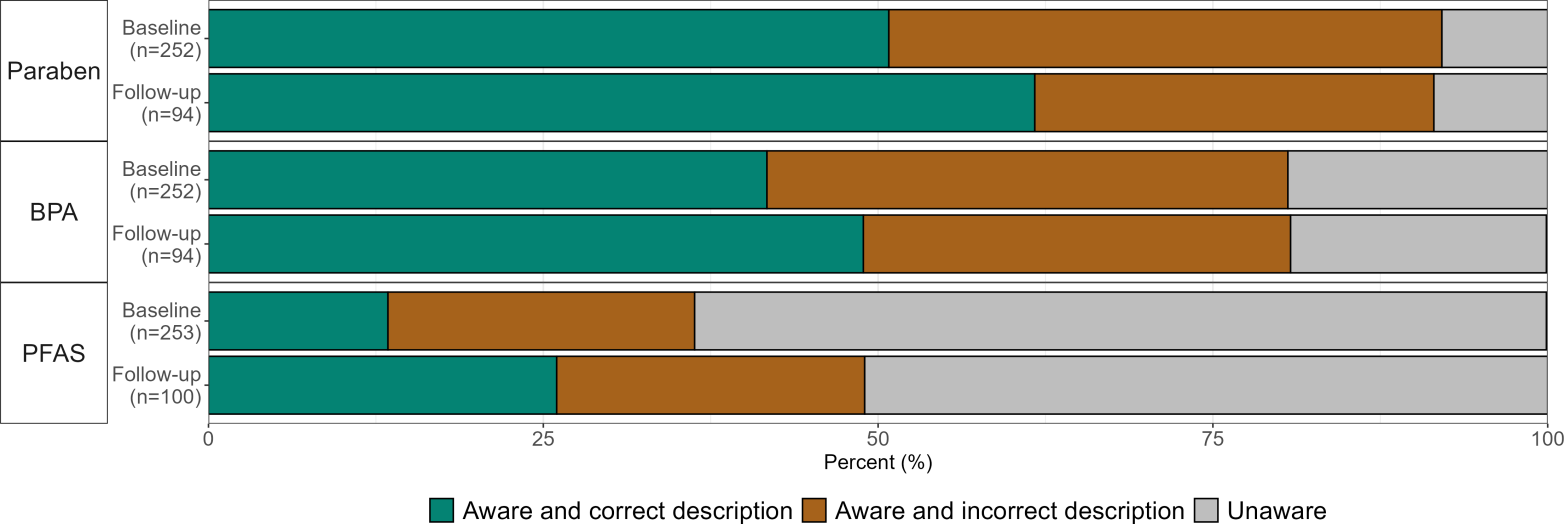
Baseline and follow-up survey responses from audiences for EDC awareness questions. For respondents who indicated that they had heard of an EDC before, they were then asked to select its description. Missing answers to the dependent question were classified as incorrect. The number of survey respondents for each question is indicated in parentheses. BPA: bisphenol A; EDC: endocrine-disrupting chemical; PFAS: per- and polyfluoroalkyl substance.

A large majority of respondents had heard of parabens at baseline (n=232, 92.1%) and follow-up (n=86, 91.5%), and more than half selected the correct description of parabens at baseline (n=128, 55.2%; Figure 5) and follow-up (n=58, 67.4%). BPA was familiar to approximately 80% of respondents, and more than 50% of respondents selected its correct description at both baseline (n=105) and follow-up (n=46). Significantly more follow-up survey respondents (n=49) than baseline respondents (n=92) reported having heard of PFAS (49% vs 36.4%; *P*=.03; Table S8 in Multimedia Appendix 1). More follow-up respondents (n=49) than baseline respondents (n=92) were able to identify the description of the PFAS (n=26, 53.1% vs n=34, 37%; *P*=.07; Table S9 in Multimedia Appendix 1).

### Audience Behaviors

More baseline respondents reported avoiding parabens and BPA at baseline (n=39, 15.3% and n=38, 14.9%, respectively, Table 3) than PFAS (n=9, 3.5%) or fragrance (n=5, 2%). A higher proportion of follow-up respondents than baseline respondents individually listed PFAS (n=17, 16.8% vs n=9, 3.5%; *P*<.001), parabens (n=33, 32.7% vs n=39, 15.3%; *P*<.001), BPA (n=25, 24.8% vs n=38, 14.9%; *P*=.03), and fragrance (n=6, 5.9% vs n=5, 2.0%; *P*=.08) as a chemical to avoid when selecting products.

**Table 3.** Comparison of baseline and follow-up survey responses who self-reported avoiding specific chemicals on ingredient labels.

Chemical	Baseline (N=255), n (%)	Follow-up (N=101), n (%)	*P* value^a^
Paraben	39 (15.3)	33 (32.7)	<.001
BPA^b^	38 (14.9)	25 (24.8)	.03
PFAS^c^	9 (3.5)	17 (16.8)	<.001
Fragrance	5 (2.0)	6 (5.9)	.08

a Fisher exact test.

b BPA: bisphenol A.

c PFAS: per- and polyfluoroalkyl substance.

Most baseline respondents (n=133, 57.1%) reported that they at least sometimes bought a product in the past year with worrisome chemicals because they could not afford a safer one. Significantly more follow-up respondents reported that they would check ingredients always or often before buying a product (n=73, 80.2% vs n=115, 46.9%; *P*<.001; Table S10 in Multimedia Appendix 1) and always or often consider a company’s policy about chemicals when deciding whether to purchase a product (n=68, 80% vs n=63, 26.8%; *P*<.001) compared to baseline respondents.

## Discussion

### Principal Findings

The POWER project demonstrated that strategic SMI communication can engage large audiences of Black women with information on EDCs in consumer products to increase knowledge and support change in behaviors. We observed greater EDC-related knowledge and awareness for both influencers (after attending a training workshop) and their audiences (after viewing an SMI campaign post). After the campaign, more respondents reported intentions to avoid EDCs, check ingredients, and consider a company’s policy about chemicals when deciding to purchase a product compared to the baseline survey. These results suggest that partnerships between nonprofit organizations and SMIs to translate research can be successfully used to deliver targeted communications about EDCs that promote informed decision-making.

We used strategic communications, informed by a CHW approach, for engaging Black women with EDC-related information by training and compensating SMIs with large and engaged audiences of Black women. Seven SMIs each created 1 Instagram post and reached a total of 16,000 accounts in a month. Since content only needs to be posted once and can be circulated indefinitely, social media posts continue to reach users without any further action by the SMI. Webinars are another venue for digital communications but frequently have less reach—over 70% of webinars have fewer than 50 live attendees to engage with content [55]. In contrast, we engaged an audience that was nearly 3 orders of magnitude larger. In our SMI communications, engagement by reach (10%) was twice as high as the average engagement rate by reach across all Instagram posts (4.55%) in 2023‐2024 [56], the year of this campaign. Seen as peers by their audience, SMIs can reach a specific audience through direct engagement with content [57] and gather more attention through audience shares [58]. This highlights the value of working with SMIs with highly engaged audiences and suggests that the content of our campaign was perceived as interesting or important among members of their audiences.

Our tailored training workshops for SMIs were effective at preparing them to be knowledge mediators about EDCs. We saw improvements in EDC knowledge, awareness, and behavior intentions among SMIs. Communal learning among Black women has been shown to be effective at sharing health information [59,60], and our results also support this. The workshop curriculum was closely aligned with our specific communication goals, which allowed us to keep the training brief (60 minutes), particularly compared to traditional CHW training. Content review by the scientific team also ensured quality control despite the short training period. The training duration will make it easier to scale the program and train more SMIs in the future. Additionally, new training modules could be developed to address other environmental health messages. Training SMIs to deliver evidence-based content can help bridge the gap between scientific research and public engagement [61].

We established collaborations with SMIs who identified as Black women to reach other Black women via a trusted messenger [62,63]. While different from the geographic community unit typically used by the CHW model, we considered the audience of an SMI to be a self-selected digital community of people with shared interests where the SMI serves as a trusted voice. Our approach is further supported by previous research suggesting that educational efforts partnering with Black SMIs may be persuasive to Black audiences [50,64]. We intentionally recruited SMIs who were not primarily situated in the environmental health social media space to reach audiences who may not already be familiar with the shared content.

Our results suggest that viewing SMI content may be effective at influencing consumer behavior around EDCs, including checking ingredients on product labels and considering a company’s policy about chemicals. However, implementing these strategies may be challenging because many respondents also reported experiencing financial constraints to buying safer products. Additionally, access to safer products may be limited based on the products available in different communities [12]. These problems underscore the importance of policy initiatives that restrict chemicals of concern and promote the design of safer products.

To our knowledge, this is the first study to train SMIs to serve as digital CHWs as part of strategic SMI communications. Other studies have used social media to effectively promote behavior change among populations with health disparities without working with SMIs [65-67]. A study conducted in Nigeria suggested that SMIs may be effective options for health promotions [68]; however, this was a theoretical analysis of popular SMIs in a region of Nigeria and discussed health generally rather than any specific issue. Few studies have educated consumers digitally about EDCs in the environment to support disease prevention. Wright et al [69] worked with “mommy bloggers” to integrate science-based messages about breast cancer and environmental risk tailored for mothers and daughters. Their approach led to higher breast cancer risk and prevention information scores for blog readers who were exposed to the intervention messages than those who were not exposed. We similarly found that respondents who saw posts with relevant content had higher EDC knowledge compared to followers who did not see the content. In contrast to our study where most respondents were Black women, Wright et al [69] reported limited reach to Black women—only 5% of people reached in that study were Black. Their focus was also on reaching blog audiences, whereas we worked on Instagram to optimize reach.

The Bench to Community Initiative is focused on communicating information about EDCs and health to Black communities to reduce breast cancer risk [70]. This community-based participatory research program shares information directly with its network through organizational programs and studies. In contrast, our social media approach facilitated engagement outside of our organizations’ direct networks to large, diffuse networks, many of which had limited previous engagement and knowledge on environmental health. This strategy allowed us to tap into existing networks of trust and credibility that SMIs have built with their followers, particularly Black women who rely on social media for product information [71,72].

### Limitations

Black women come from many backgrounds, ethnicities, community contexts, and lived experiences. As such, this work may not be generalizable to all Black women. Further, we did not evaluate any distinctions with those who identified as multiracial. Our goal was to measure the effect of SMI communications on EDC knowledge, awareness, and behavior intentions, so we did not focus on traditional indicators of influence or broader impact among the SMIs to quantify the full reach of our campaign. As is standard for the industry, we compensated the SMIs for their work on this project. However, because of budget limitations, compensation was below the typical asking rate of some SMIs, and we hypothesize that SMIs were also motivated to participate because of their interest in the project goals. Increased compensation would likely allow us to work with SMIs who have larger audiences or, potentially, to support ongoing partnerships. Some of the SMIs involved have also been compensated by consumer product brands to promote their products, which is a typical business model for SMIs. To limit potential conflicts of interest, the posts developed for the POWER project communications did not endorse any specific brands or companies.

The SMIs and survey respondents in our study reported higher levels of formal education than the US average, which may contribute to the high baseline knowledge levels we observed [73]. We also could not track whether survey respondents saw content from multiple SMIs. As we limited our surveys to fewer than 20 questions, there are additional aspects of environmental health literacy that were not explored. Although we asked for contact information to track individual respondents from baseline to follow-up, most respondents did not provide their information, limiting our ability to analyze individual-level changes and effect modification by demographic characteristics. We also could not account for secular trends (eg, news media on PFAS contamination), which may have led to overall higher EDC knowledge in the follow-up survey.

### Future Research

Bidirectional communication on social media may yield high engagement with EDC and other health-related information, where SMIs respond to questions under content and share multiple posts. Future research should explore the long-term impact of such strategic SMI communication on health-related behaviors and experimentally assess the added value of using SMIs compared to other outlets. Furthermore, studies should consider evaluating measurable changes in EDC exposures. Sustaining partnerships beyond individual projects will support more consistent and widespread sharing of evidence-based health information to diverse communities. Communications that connect with established social media accounts can leverage existing audiences and integrate evidence-based information into spaces where Black women are already engaged, increasing the reach and impact of the health promotion efforts. These partnerships could also be considered by other actors interested in promoting health-related messages, including academic and medical institutions.

### Policy Implications

The POWER project was undertaken in part because existing policies have fallen short in protecting Black women from harmful exposures to EDCs in consumer products. Despite frameworks like the Federal Food, Drug, and Cosmetic Act (1938) [74] and the Toxic Substances Control Act (1976) [75], many chemicals, including EDCs and others linked to serious health outcomes, continue to enter the market without sufficient safety review or labeling requirements. Though recent efforts such as the Modernization of Cosmetics Regulation Act (2022) have expanded Food and Drug Administration oversight, major gaps remain, particularly around premarket safety assessment and transparency [76]. These regulatory shortcomings are further compounded by recent efforts to weaken environmental justice protections, including challenges to Executive Order 12898, which historically directed federal agencies to address environmental harms in marginalized communities [77]. In light of these regulatory gaps, the POWER project aimed to educate people about how to make informed consumer decisions (including limitations of current public policy), and our results support the efficacy of our SMI-based approach. Complementary regulatory actions, such as mandatory labeling of EDCs in consumer products, would empower communities with greater transparency. Strengthening both policy and public engagement will be essential to reduce exposure disparities and support informed decision-making among Black women.

### Conclusions

Our results demonstrate that strategic SMI communication is an effective approach to widely sharing information about EDCs with Black women. As trusted sources of information, SMIs have a unique voice in their communities and can act as knowledge mediators like CHWs. By collaborating with SMIs, researchers can help shape health promotion campaigns to inform audiences who are harder to reach through traditional communication channels, including Black women and other populations affected by health disparities. The methods developed in this study are not limited to environmental health and can be applied broadly to other health promotion efforts.

## Supplementary material

10.2196/66128 Multimedia Appendix 1 Supporting tables and figures, including detailed survey information and expanded analyses referenced in the main text.

## ABBREVIATIONS

BPA: bisphenol A
CHW: community health worker
EDC: endocrine-disrupting chemical
PFAS: per- and polyfluoroalkyl substance
POWER: Product Options in Women-Engaged Research
SMI: social media influencer
