# Supplementary material for: The Power of Strategic Social Media Influencer Communication to Improve Black Women’s Knowledge and Awareness of Environmental Endocrine-Disrupting Chemicals: Surveys of Instagram Users
Source: J Med Internet Res. 2025 Jul 25;27:e66128. doi: 10.2196/66128 (PMC12296211; doi:10.2196/66128)
Supplement: Multimedia Appendix 1 [file jmir-v27-e66128-s001.docx]

Table S1. Knowledge and endocrine disrupting compound (EDC) awareness survey questions, the correct answer to the questions, suggested talking points shared at the social media influencer (SMI) training workshop, and the number of SMIs (N = 7) who covered the topic in their social media content.

| Question | Question Type | Correct Answer | Talking points | Mentioned in content (n) |
| --- | --- | --- | --- | --- |
| Everyday products—like shampoo, sofas, and plastic bottles—sometimes contain chemicals that can upset the balance of hormones in a person’s body. | Knowledge | True | Chemicals that disrupt a person’s hormones are in everyday products including cosmetics, furniture, cookware, and plastic food and drink containers. | 7 |
| Chemicals have to pass many safety tests before they can be used in products in the U.S. | Knowledge | False | Chemicals do not have to pass many safety tests before they are used in US products. | 5 |
| Companies are required to tell consumers all the chemicals they put in household goods like paint, pots and pans, or carpet. | Knowledge | False | Companies are not required to tell consumers all the chemicals that they use in products.  Manufacturers are not required to list their fragrance ingredients. | 4 |
| Tests by the U.S. Centers for Disease Control and Prevention (CDC) show that everyone has chemicals from the environment detected in their body. | Knowledge | True | The CDC has found consumer product chemicals in everyone’s body | 2 |
| A product labeled “BPA-free” will not contain any toxic chemicals. | Knowledge | False |  | 0 |
| Reading the label will tell you all of the chemicals in household cleaning products. | Knowledge | False | Fragrances added to products may include a combination of toxic chemicals. | 0 |
| Have you heard of parabens before? | EDC awareness | -- | -- | 6 |
| Which of these statements is true for parabens? | EDC awareness | Parabens are added to cosmetics to keep them from spoiling. | Parabens are added to personal care products as preservatives. | 2 |
| Have you heard of BPA (bisphenol A) before? | EDC awareness | -- | -- | 7 |
| Which of these statements is true for BPA (bisphenol A)? | EDC awareness | BPA is used in polycarbonate plastic, receipt paper, and canned food linings. | Bisphenol A (BPA) is a hormone-disrupting chemical added to plastic to make it more durable. | 3 |
| Have you heard of PFAS (per- and polyfluoroalkyl substances) before? | EDC awareness | -- | -- | 5 |
| Which of these statements is true for PFAS (per- and polyfluoroalkyl substances)? | EDC awareness | PFAS are used to make things non-stick, waterproof, or grease resistant. | PFAS is a class of chemicals added to products to make the products non-stick, water-proof, or resistant to oil and grease. | 3 |

Table S2. Behavior survey questions asked in the baseline and follow-up surveys.

| Baseline | Follow-up |
| --- | --- |
| How often do you check a product label for chemical ingredients before you buy it? | Now that you’ve seen the post, how often will you check a product label for chemical ingredients before you buy it? |
| What chemicals do you try to avoid by reading the product label? Don’t worry about spelling the chemicals correctly. | What chemicals will you try to avoid by reading the product label? Don’t worry about spelling the chemicals correctly. |
| In the past year, how often did you consider a company’s policy about chemicals when deciding whether to purchase from a particular brand or business? | After seeing this post, how often will you now consider a company’s policy about chemicals when deciding whether to purchase from a particular brand or business? |
| In the past year, how often did you buy a product with worrisome chemicals because you couldn’t afford a safer one? | -- |

Table S3. Outline of training workshop topics.

| **Topics** |
| --- |
| - Survey influencer introduction - RSP and Silent Spring introduction - Influencer introduction - Share deliverables - EDCs in everyday products - How chemicals enter the body - Exposure disparities and differences in product use - Chemicals of concern found in products used by Black women - Tips for reducing exposure - Regulatory oversight - Social media content ideas - Resources - Expected project outcomes - Remaining deliverables |

Table S4: Influencers, their niche, Instagram follower count, and a link to the content.

| **Influencer Handle** | **Niche** | **Instagram followers** | **Content Link** |
| --- | --- | --- | --- |
| @plantbasedbre | Food & Lifestyle | 54,800+ | https://www.instagram.com/p/Cy32xe8pwYe/?utm_source=ig_web_copy_link&igshid=MzRlODBiNWFlZA== |
| @hearhairr | Hair & Beauty | 52,500+ | https://www.instagram.com/reel/CygisnYOW66/?igshid=MzRlODBiNWFlZA%3D%3D |
| @beeharris | Art & Lifestyle | 14,600+ | https://www.instagram.com/p/CygUXIJOmK-/?utm_source=ig_web_copy_link&igshid=MzRlODBiNWFlZA== |
| @brialleringer | Sustainability & Lifestyle | 10,500+ | https://www.instagram.com/reel/Cy_z4xcvolg/?igshid=MzRlODBiNWFlZA== |
| @iamcamillesmith | Beauty & STEM | 8,969 | https://www.instagram.com/reel/CyyRW9CuNyq/?igshid=MzRlODBiNWFlZA== |
| @nellynelle19 | Wellness & Lifestyle | 23,000+ | https://www.instagram.com/reel/Cy0dNEWOcHA/?utm_source=ig_web_copy_link&igshid=MzRlODBiNWFlZA== |
| @lashannontaylor | Fashion & Lifestyle | 5,930 | https://www.instagram.com/reel/CyoJ1ZWAhXf/?utm_source=ig_web_copy_link |

Table S5: Number and percent of correct responses to baseline EDC knowledge questions by self-identified demographic group. Significant (p<0.05) differences in proportion correct by demographic group in bold.

|  | **Black Woman** | | | **Race** | | | **Gender** | | | **Education** | | |
| --- | --- | --- | --- | --- | --- | --- | --- | --- | --- | --- | --- | --- |
| **Question** | **Yes** N = 164*^1^* | **No** N = 53*^1^* | **p-value***^2^* | **Black** N = 174*^1^* | **Not Black** N = 44*^1^* | **p-value***^2^* | **Woman** N = 211*^1^* | **Not Woman** N = 14*^1^* | **p-value***^2^* | **Associate or less** N = 52*^1^* | **Bachelor's or more** N = 176*^1^* | **p-value***^2^* |
| Everyday products-like shampoo, sofas, and plastic bottles-sometimes contain chemicals that can upset the balance of hormones in a person's body. | **118 (72.0%)** | **29 (54.7%)** | **0.03** | **125 (71.8%)** | **22 (50.0%)** | **0.01** | 144 (68.2%) | 8 (57.1%) | 0.39 | 35 (67.3%) | 119 (67.6%) | 1.0 |
| Tests by the U.S. Centers for Disease Control and Prevention (CDC) show that everyone has chemicals from the environment detected in their body. | 83 (50.6%) | 23 (43.4%) | 0.43 | 87 (50.0%) | 19 (43.2%) | 0.50 | 104 (49.3%) | 6 (42.9%) | 0.78 | 21 (40.4%) | 91 (51.7%) | 0.16 |
| A product labeled BPA-free will not contain any toxic chemicals. | 57 (34.8%) | 18 (34.0%) | 1.0 | 60 (34.5%) | 15 (34.1%) | 1.0 | 72 (34.1%) | 4 (28.6%) | 0.78 | 13 (25.0%) | 64 (36.4%) | 0.14 |
| Chemicals have to pass many safety tests before they can be used in products in the U.S. | 30 (18.3%) | 10 (18.9%) | 1.0 | 31 (17.8%) | 9 (20.5%) | 0.67 | 42 (19.9%) | 1 (7.1%) | 0.48 | 8 (15.4%) | 36 (20.5%) | 0.55 |
| Companies are required to tell consumers all the chemicals they put in household goods like paint, pots and pans, or carpet. | 42 (25.6%) | 16 (30.2%) | 0.59 | 46 (26.4%) | 12 (27.3%) | 1.0 | 56 (26.5%) | 4 (28.6%) | 1.0 | 9 (17.3%) | 53 (30.1%) | 0.08 |
| Reading the label will tell you all of the chemicals in household cleaning products. | 52 (31.7%) | 17 (32.1%) | 1.0 | 54 (31.0%) | 15 (34.1%) | 0.72 | 67 (31.8%) | 3 (21.4%) | 0.56 | 11 (21.2%) | 61 (34.7%) | 0.09 |
| *^1^*n (%) | | | | | | | | | | | | |
| *^2^*Fisher's exact test | | | | | | | | | | | | |

Table S6. Summary of correct responses to baseline and follow up surveys.

| **Question** | **Correct answer** | **Baseline**,  N = 255*^a^* | **Follow-up**, N = 101*^a^* | **p-value***^b^* |
| --- | --- | --- | --- | --- |
| Everyday products-like shampoo, sofas, and plastic bottles-sometimes contain chemicals that can upset the balance of hormones in a person's body.*^c^* | TRUE | 173 (67.8%) | 81 (80.2%) | 0.02 |
| Tests by the U.S. Centers for Disease Control and Prevention (CDC) show that everyone has chemicals from the environment detected in their body. | TRUE | 122 (47.8%) | 57 (56.4%) | 0.16 |
| A product labeled BPA-free will not contain any toxic chemicals.*^d^* | FALSE | 88 (34.5%) | 31 (30.7%) | 0.53 |
| Chemicals have to pass many safety tests before they can be used in products in the U.S. | FALSE | 49 (19.2%) | 26 (25.7%) | 0.19 |
| Companies are required to tell consumers all the chemicals they put in household goods like paint, pots and pans, or carpet. | FALSE | 66 (25.9%) | 32 (31.7%) | 0.29 |
| Reading the label will tell you all of the chemicals in household cleaning products.*^d^* | FALSE | 80 (31.4%) | 30 (29.7%) | 0.80 |
| *^a^*n (%)  *^b^*Fisher's exact test  *^c^*Question was mentioned by all the social media influencers  *^d^*Question was mentioned by all the social media influencers | | | | |

| **Question** | **Correct answer** | **Nonviewers***^a^* | **Viewers***^b^* | **p-value***^c^* |
| --- | --- | --- | --- | --- |
| Chemicals have to pass many safety tests before they can be used in products in the U.S.*^d^* | FALSE | 6 (18.8%) | 20 (29.0%) | .33 |
| Tests by the U.S. Centers for Disease Control and Prevention show that everyone has chemicals from the environment detected in their body.*^e^* | TRUE | 54 (56.8%) | 3 (50.0%) | 1.0 |
| Companies are required to tell consumers all the chemicals they put in household goods like paint, pots and pans, or carpet.*^f^* | FALSE | -- | -- | -- |

Table S7. Summary of correct responses to follow-up survey questions from respondents who did not view (nonviewers) or who did view (viewers) a social media post containing associated content.

*^a^*Nonviewer *N* varies for each question, n (%)

*^b^*Viewer *N* varies for each question, n (%)

*^c^*p-value calculated using Fisher’s exact test

*^d^*Nonviewers N = 32, Viewer N = 69

*^e^*Nonviewers N = 95, Viewer N = 6

*^f^*Viewers and nonviewer could not be distinguished for this question

Table S8. Comparison of responses to baseline and follow-up survey questions for respondents who reported awareness of endocrine disrupting compounds.

| **Chemical** | **Baseline***^a^* | **Follow-up***^b^* | **p-value***^c^* |
| --- | --- | --- | --- |
| Parabens*^d^* | 232 (92.1%) | 86 (91.5%) | .82 |
| Bisphenol A*^d^* | 203 (80.6%) | 76 (80.9%) | >.99 |
| Per- and polyfluoroalkyl substances*^e^* | 92 (36.4%) | 49 (49.0%) | .03 |

*^a^*Baseline *N* varies for each question, n (%)

*^b^*Follow-up *N* varies for each question, n (%)

*^c^*p-value calculated using Fisher’s exact test

*^d^*Baseline N = 252, Follow-up N = 94

*^e^*Baseline N = 253, Follow-up N = 100

Table S9. Comparison of correct baseline and follow-up survey responses for description of endocrine disrupting compounds. Respondents were prompted to select the description only if they answered that they were aware of the endocrine disrupting compound.

| **Chemical** | **Baseline***^a^* | **Follow-up***^b^* | **p-value***^c^* |
| --- | --- | --- | --- |
| Parabens*^d^* | 128 (55.2%) | 58 (67.4%) | .055 |
| Bisphenol A*^e^* | 105 (51.7%) | 46 (60.5%) | .22 |
| Per- and polyfluoroalkyl substances*^f^* | 34 (37.0%) | 26 (53.1%) | .07 |

*^a^*Baseline *N* varies for each question, n (%)

*^b^*Follow-up *N* varies for each question, n (%)

*^c^*p-value calculated using Fisher’s exact test

*^d^*Baseline N = 232, Follow-up N = 86

*^e^*Baseline N = 203, Follow-up N = 76

*^f^*Baseline N = 92, Follow-up N = 49

Table S10. Comparison of baseline and follow-up survey responses for self-reported “often” or “always” to behaviors related to consumer product choices.

| **Behavior** | **Baseline***^a^* | **Follow-up***^b^* | **p-value***^c^* |
| --- | --- | --- | --- |
| Check a product label for chemical ingredients before you buy it*^d^* | 115 (46.9%) | 73 (80.2%) | <.001 |
| Consider a company's policy about chemicals when purchasing*^e^* | 63 (26.8%) | 68 (80.0%) | <.001 |

*^a^*Baseline *N* varies for each question, n (%)

*^b^*Follow-up *N* varies for each question, n (%)

*^c^*p-value calculated using Fisher’s exact test

*^d^*Baseline N = 245, Follow-up N = 91

*^e^*Baseline N = 235, Follow-up N = 85


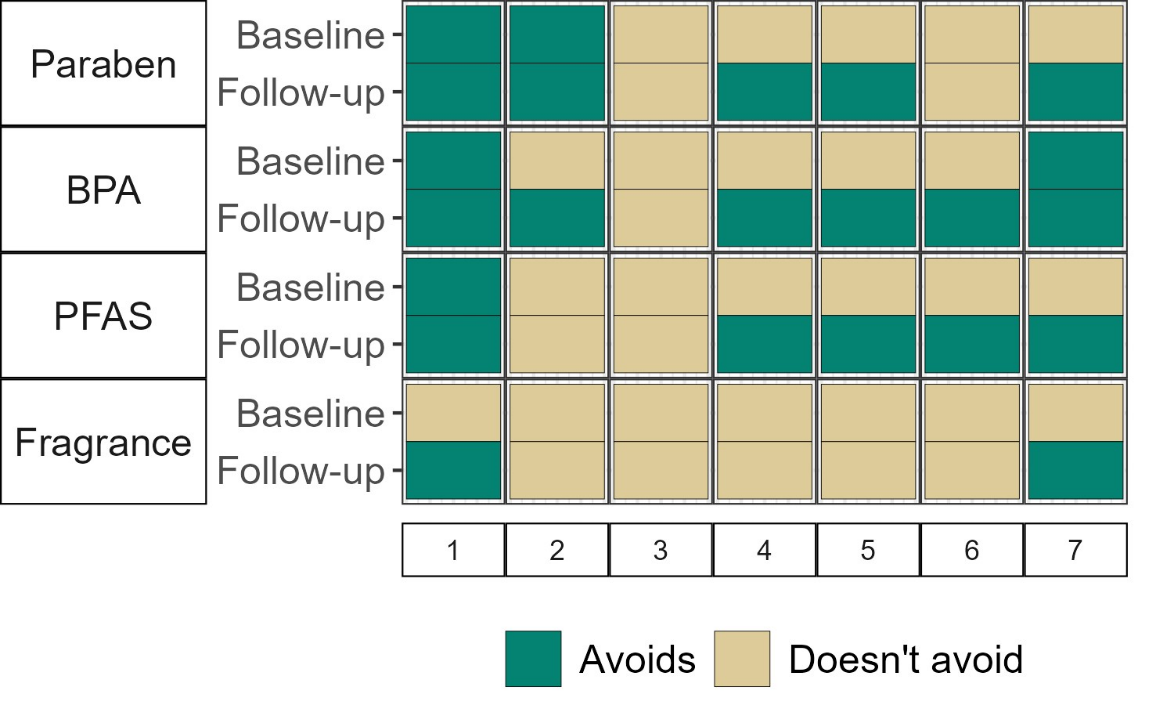


Fig. S1. Social media influencers’ (N = 7) reported avoidance of specific chemicals on ingredient labels at baseline and follow-up.


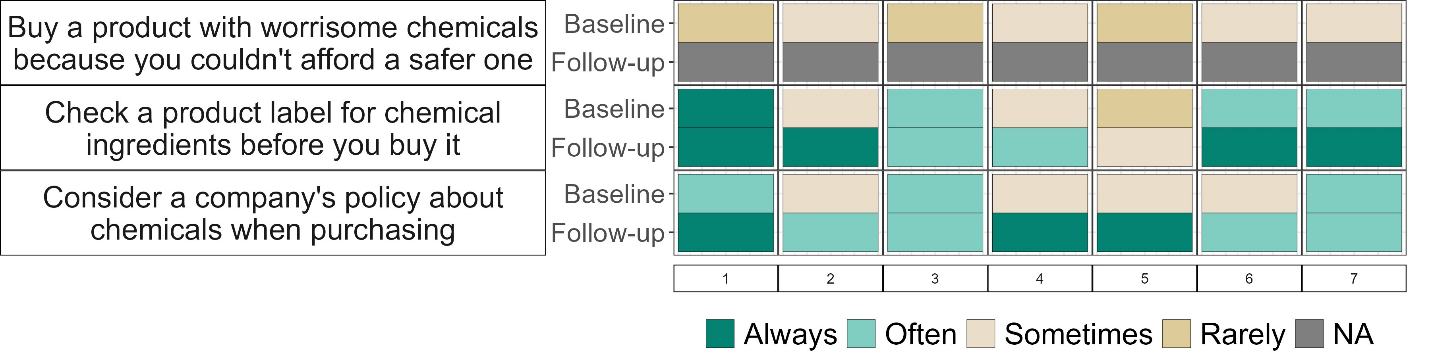


Fig. S2. Baseline and follow-up survey responses of social media influencers for environmental health literacy behavior questions. At baseline, influencers were asked about behavior in the past year. At follow-up, influencers were asked how likely they would be to perform the behaviors in the future.
